# Prospective pilot study of functional assessment of the Sphincter of Oddi via cine-dynamic MRCP with selective inversion recovery pulse

**DOI:** 10.1007/s00535-026-02344-1

**Published:** 2026-01-22

**Authors:** Yuki Oka, Arata Sakai, Atsuhiro Masuda, Keitaro Sofue, Shigeto Ashina, Takashi Kobayashi, Masahiro Tsujimae, Masanori Gonda, Noriko Inomata, Mika Miki, Yoshiyuki Harada, Noriko Juri, Yosuke Irie, Tetsuhisa Ko, Yusuke Yokotani, Akira Shirohata, Kaoruko Kanamaru, Takafumi Tokunaga, Kenta Yamamoto, Kohei Okamoto, Kento Ogawa, Yuta Kawase, Tatsuya Kageyama, Ryuji Shimada, Yuichiro Somiya, Kentaro Nishiuchi, Norimitsu Uza, Yuzo Kodama

**Affiliations:** 1 Division of Gastroenterology, Department of Internal Medicine, Kobe University Graduate School of Medicine, Kobe, 650-0017 Japan; 2 Department of Radiology, Kobe University Graduate School of Medicine, Kobe, 650-0017 Japan; 3 Department of Gastroenterology, Akashi Medical Center, 743-33 Yagi, Ookubo-Cho, Akashi, Hyogo 674-0063 Japan; 4 Center for Radiology and Radiation Oncology, Kobe University Hospital, Kobe, 650-0017 Japan

**Keywords:** Sphincter of Oddi dysfunction, Idiopathic pancreatitis, Endoscopic sphincterotomy, Magnetic resonance cholangiopancreatography, Sphincter of Oddi manometry

## Abstract

**Background:**

Sphincter of Oddi dysfunction (SOD) can cause unexplained biliary pain and idiopathic pancreatitis. Although Rome IV criteria recommend sphincter of Oddi manometry (SOM) for diagnosis, SOM is invasive and carries pancreatitis risk. We hypothesized that cine-dynamic magnetic resonance cholangiopancreatography (MRCP) could non-invasively visualize bile and pancreatic juice flow, enabling functional papillary assessment.

**Methods:**

In this prospective observational study, 40 participants were enrolled, and 29 were included in the final analysis after excluding 11 participants who did not meet the Rome IV criteria (10 healthy controls, 7 with suspected biliary-type SOD [BSOD], and 12 with suspected pancreatic-type SOD [PSOD]). Cine-dynamic MRCP was performed with 20 sequential frames over 5 min. Two quantitative indices were assessed: flow frequency and secretion grade (distance traveled by bile or pancreatic juice).

**Results:**

Bile flow frequency and secretion grade were significantly lower in both BSOD and PSOD than in controls: frequency (median [range], 13.5 [6–19] in controls vs. 2.0 [1–17] in BSOD, *p* = 0.006; vs. 8.0 [3–14] in PSOD, *p* = 0.008) and secretion grade (1.6 [0.3–2.05] in controls vs. 0.2 [0.1–1.3] in BSOD, *p* = 0.001; vs. 0.5 [0.15–1.75] in PSOD, *p* = 0.03). Pancreatic juice flow showed no significant difference between BSOD and controls but was significantly reduced in PSOD: frequency (16 [14–19] in controls vs. 9.5 [4–17] in PSOD, *p* < 0.001) and secretion grade (2.15 [0.7–3.25] in controls vs. 0.98 [0.25–2.9] in PSOD, *p* = 0.003). Cine-dynamic MRCP parameters improved after sphincterotomy in six patients.

**Conclusions:**

Cine-dynamic MRCP enables non-invasive visualization and quantification of bile and pancreatic juice flow, providing functional assessment of the sphincter of Oddi.

**Supplementary Information:**

The online version contains supplementary material available at 10.1007/s00535-026-02344-1.

## Introduction

Sphincter of Oddi (SO) dysfunction (SOD) is characterized by hypercontraction of the sphincter, leading to impaired outflow of bile and pancreatic juice. This functional obstruction results in elevated pressure within both the biliary and pancreatic ducts, potentially leading to various pathological conditions. Several etiologies have been proposed, including abnormal sensitivity to cholecystokinin—a gastrointestinal hormone involved in gallbladder contraction and SO relaxation, as well as dysregulation of nociceptive pain pathways [1, 2].

Within the diagnostic framework for functional gastrointestinal disorders, SOD is categorized as a distinct entity under the classification “Gallbladder and Sphincter of Oddi Disorders” in the Rome criteria. The most recent version, Rome IV, published in 2016, classifies SOD into two subtypes: biliary-type and pancreatic-type SOD (BSOD and PSOD, respectively) [3]. According to the Rome IV criteria, BSOD is diagnosed based on a clinical history consistent with biliary pain, absence of choledocholithiasis or other structural diseases, and either elevated liver enzymes or bile duct dilatation on imaging. Supportive diagnostic tools, such as sphincter of Oddi manometry (SOM) and hepatobiliary scintigraphy, are also referenced. For PSOD, the Rome IV criteria include: (1) history of recurrent episodes of acute pancreatitis, (2) no other identifiable causes of pancreatitis, (3) no structural abnormalities identified on endoscopic ultrasonography, and (4) abnormal SOM findings. SOM remains the only direct method for measuring SO pressure, but it carries a substantial risk of post-endoscopic retrograde cholangiopancreatography pancreatitis (PEP), with an incidence of 4–10% [4]. Our previous study demonstrated that SOM using a guidewire-type manometer is a safe and reliable technique for reducing the incidence of PEP, although a small proportion of patients still developed pancreatitis [5]. Therefore, there remains a need for a safer and non-invasive method for evaluating SO function.

Cine-dynamic magnetic resonance cholangiopancreatography (MRCP) with spatially selective inversion recovery (IR) pulses has emerged as a promising non-invasive modality for visualizing the physiological flow of pancreatic juice and bile [6, 7]. Previous studies have highlighted the utility of cine-dynamic MRCP for the early diagnosis and severity assessment of chronic pancreatitis, and its correlation with the N-benzoyl-L-tyrosyl-*p*-aminobenzoic acid test has been demonstrated [8, 9]. However, to date, no study has investigated its potential for evaluating SOD or for directly assessing sphincter function.

Based on this background, we hypothesized that cine-dynamic MRCP with spatially selective IR pulses could serve as a non-invasive imaging modality for assessing SO function. To test this hypothesis, we investigated alterations in bile and pancreatic juice flow patterns in patients with BSOD and PSOD. Furthermore, given that endoscopic sphincterotomy (EST) is recommended by the Rome IV criteria and has been validated as an effective treatment for SOD [10, 11], we assessed changes in cine-dynamic MRCP parameters before and after EST.

## Methods

### Study design

This single-center prospective observational study was conducted in accordance with the declaration of Helsinki, approved by the ethics committee of Kobe university hospital (No. A190014), and registered with the UNIVERSITY HOSPITAL medical information network–clinical trials registry (UMIN–CTR) (UMIN000036686). Written informed consent was obtained from all participants before cine-dynamic MRCP.

### Study population

This study included consecutive patients who underwent cine-dynamic MRCP between October 2019 and September 2024 for the evaluation of unexplained biliary pain or idiopathic recurrent acute pancreatitis (IRAP), as well as healthy volunteers with no history of pancreatobiliary disease or regular medication use. All healthy controls underwent annual medical checkups, including physical examination and laboratory tests, by board-certified physicians. No abnormalities were identified at the time of enrollment, minimizing the risk of subclinical or undiagnosed conditions. Biliary pain was defined according to the Rome IV criteria (Online Resource 1). Idiopathic pancreatitis was diagnosed based on a comprehensive clinical assessment, including medical history (e.g., medication use, family history of pancreatitis, and history of trauma) and physical examination. In addition, at least 2 imaging modalities—contrast-enhanced abdominal computed tomography (CT), MRCP, or endoscopic ultrasound—were required to rule out identifiable causes. Acute pancreatitis was diagnosed using standard clinical criteria, excluding other pancreatic and acute abdominal conditions, and requiring the presence of at least 2 of the following: (1) acute pain and tenderness in the upper abdomen; (2) elevated pancreatic enzyme levels in blood, urine, or ascitic fluid; and (3) imaging findings consistent with acute pancreatitis on ultrasonography, CT, or magnetic resonance imaging (MRI) [12].

Both the control and patient groups were subjected to specific exclusion criteria. Individuals who were unable to undergo MRI because of contraindications, such as metallic implants or tattoos, were excluded. In addition, individuals with known hypersensitivity to manganese chloride tetrahydrate—an oral contrast agent used in MRCP—were excluded from the study. Patients with biliary pain and those with idiopathic recurrent pancreatitis who did not meet the Rome IV criteria for SOD (Online Resource 2 and 3) were excluded and classified into the BSOD-suspected and PSOD-suspected groups, respectively.

### MRI technique

MRI was performed using a 3.0-T MR system (Vantage Centurian; Canon medical systems, Tochigi, Japan) equipped with phased-array coils (16 channels with 32 elements) and bed-embedded spine coils (32 channels with 40 elements). Participants fasted for at least 5 h before the examination, during which only clear water was permitted. Medications that could potentially affect sphincter of Oddi or papillary motility—such as nifedipine, trimebutine, duloxetine, H_2_ blockers, nitrates, and anticholinergic agents—were withheld for at least 24 h before imaging. Immediately before MRI, participants orally ingested 36 mg of manganese chloride tetrahydrate (in a 250 mL package of bothdel oral solution 10; Kyowa Hakko Kirin, Tokyo, Japan) to suppress bowel signal. Cine-dynamic MRCP with a spatially selective IR pulse was incorporated into the pancreatobiliary MRI protocol. Initially, a 2-dimensional (2D) thick-slab MRCP image with fat suppression was acquired during a single breath-hold using a fast advanced spin-echo sequence to visualize the main pancreatic duct in the oblique coronal and transverse planes. Imaging parameters for these sequences were as follows: repetition time (TR)/echo time (TE) = 3000/600 ms, echo train spacing = 5.0 ms, echo train length = 164, slice thickness = 40 mm, matrix = 352 × 400, field of view (FOV) = 30 × 25 cm, bandwidth = 651 Hz, parallel imaging factor = 2, fat suppression pulse type = SPAIR, and number of acquisitions = 1. Using these 2D MRCP images as reference, a spatially selective IR pulse with a width of 20 mm was applied to the pancreatic head, as perpendicularly as possible to the duct axis, to observe the movement of pancreatic juice and bile. The IR slab was positioned just above the papilla to include both the distal common bile duct and the terminal main pancreatic duct within the same slice, enabling simultaneous assessment of bile and pancreatic juice flow. Imaging parameters for cine-dynamic MRCP were as follows: TR/TE = 5000/500 ms, echo train spacing = 6.5 ms, echo train length = 157, slice thickness = 40 mm, matrix = 320 × 320, FOV = 32 × 32 cm, bandwidth = 558 Hz, parallel imaging factor = 2, fat suppression pulse type = SPAIR, number of acquisitions = 1, deep learning image reconstruction = AiCE, and selective IR pulse triggered by pulse-wave synchronization. Image acquisition was performed with a fixed timing protocol consisting of a 5 s scan period followed by a 10 s rest period, independent of patient’s heart rate. A 20 mm-wide spatially selective IR pulse was then applied perpendicular to the lower common bile duct and main pancreatic duct to observe the movement of the pancreatic juice and bile. Based on previous studies, an inversion time of 2200 ms and inversion pulse flip angle of 240° were selected to nullify the signal from the static pancreatic juice and bile [6–9]. MRCP with a spatially selective IR pulse, using the same sequence, was acquired in 5 s during breath-holding. All participants were instructed to initiate breath-holding at the end of expiration. In this technique, static pancreatic juice and bile within the area of the spatially selective IR pulse appear dark due to signal nullification. In contrast, when pancreatic juice or bile is secreted and flows, the inflow appears as high signal intensity within the IR pulse area, as the fully magnetized fluid enters the IR pulse region from the caudal side of the pancreatic duct or common bile duct. MRCP with a spatially selective IR pulse was repeated every 15 s (scanning: 5 s, rest: 10 s) over a total duration of 5 min to produce 20 cine-dynamic MRCP images [12]. An examination video obtained using cine-dynamic MRI is provided in Online Resource 4. A detailed summary of the cine-dynamic MRCP acquisition parameters and minimum technical requirements for reproducibility across MRI systems is provided in Online Resource 5.

### Image analysis

Cine-dynamic MRCP images were independently evaluated by 2 gastroenterologists (Y.O. and M.G., with 12 and 14 years of clinical experience, respectively), who were blinded to all clinical information. Any discrepancies in interpretation were resolved by consensus. In accordance with previous reports, two evaluation parameters were defined: inflow frequency and secretion grade. An inflow event was defined on a per frame basis as the appearance of a new high-intensity column of pancreatic or biliary fluid within the pre-saturation IR slab. To enhance reproducibility, a region was considered inflow-positive when a visually discernible signal increase (typically corresponding to a local rise of ≥ 10% or ≥ 1.5 SD above the surrounding parenchyma) extended continuously for ≥ 1 mm along the ductal axis. A bolus visible across consecutive frames was counted once per frame, and the total frequency was expressed as the number of frames showing inflow (maximum = 20). Secretion grade was defined by the distance traveled by the moving fluid column in the main pancreatic duct or common bile duct, using a five-point scale: grade 0 = no flow; grade 1 < 5 mm; grade 2 = 5–10 mm; grade 3 = 11–15 mm; and grade 4 > 15 mm [6] (Online Resources 6–8).

### EST procedure

All procedures were performed using a standard duodenoscope (TJF-290; olympus medical systems, Tokyo, Japan). A standard pull-type papillotome was used for biliary sphincterotomy. Medium-to-large-sized incisions were made based on the size of the duodenal papilla and at the discretion of the endoscopist. The incision was limited to the biliary orifice, and endoscopic pancreatic sphincterotomy (EPST) was not performed in any case. No adjunctive endoscopic papillary balloon dilation or stent placement was performed during the same session. Adverse events were defined and graded according to the consensus criteria proposed by Cotton et al. [13] and the subsequent ASGE lexicon update [14]. Cine-dynamic MRCP for post-procedural functional assessment was performed between 7 and 60 days after EST under the same imaging protocol as used pre-procedurally. Recurrence was defined as the development of either recurrent pancreatitis or biliary-type pain, as documented in the patients’ medical records.

### Statistical analyses

All statistical analyses were performed using EZR (Saitama Medical Center, Jichi Medical University, Saitama, Japan), a graphical user interface for R (R Foundation for Statistical Computing, Vienna, Austria). More precisely, it is a modified version of the R Commander, designed to include statistical functions frequently used in biostatistics. Data are presented as the median (range). Fisher’s exact test, Kruskal–Wallis test, and Mann–Whitney *U* test were used for between-group comparisons. For multiple pairwise comparisons following the Kruskal–Wallis test, Dunn’s post hoc test with Bonferroni adjustment was applied, and Hodges–Lehmann median differences with 95% confidence intervals (CIs) were calculated to provide effect size estimates. For paired comparisons (pre and post-EST), the Wilcoxon signed-rank test was used. To evaluate inter-reader reproducibility, inter-observer agreement for bile and pancreatic flow frequency was assessed using the single-measure, 2-way intraclass correlation coefficient (ICC[A,1]). In addition, inter-observer agreement for secretion grade was assessed using the quadratic weighted kappa (*κ*) statistic, which accounts for the degree of disagreement for ordinal variables. *κ* values were interpreted as follows: 0.21–0.40, fair agreement; 0.41–0.60, moderate agreement; 0.61–0.80, substantial agreement; and 0.81–1.00, almost perfect agreement. To account for potential confounding by age, a sensitivity analysis was performed, including only participants aged ≤ 65 years, comparing cine-dynamic MRCP parameters between the control group and patients with PSOD. Multivariate linear regression analysis was performed to evaluate the independent associations of age and clinical group (BSOD and PSOD) with the main functional imaging outcomes measured using cine-dynamic MRCP: bile flow frequency, bile secretion grade, pancreatic juice flow frequency, and pancreatic juice secretion grade. The model included age (as a continuous variable) and indicator variables for BSOD and PSOD, using the control group as the reference. Unstandardized regression coefficients (*β*), 95% CIs, and *p* values were reported. All statistical tests were two-tailed, and statistical significance was defined as *p* < 0.05.

## Results

### Patient characteristics

**Fig. 1:**
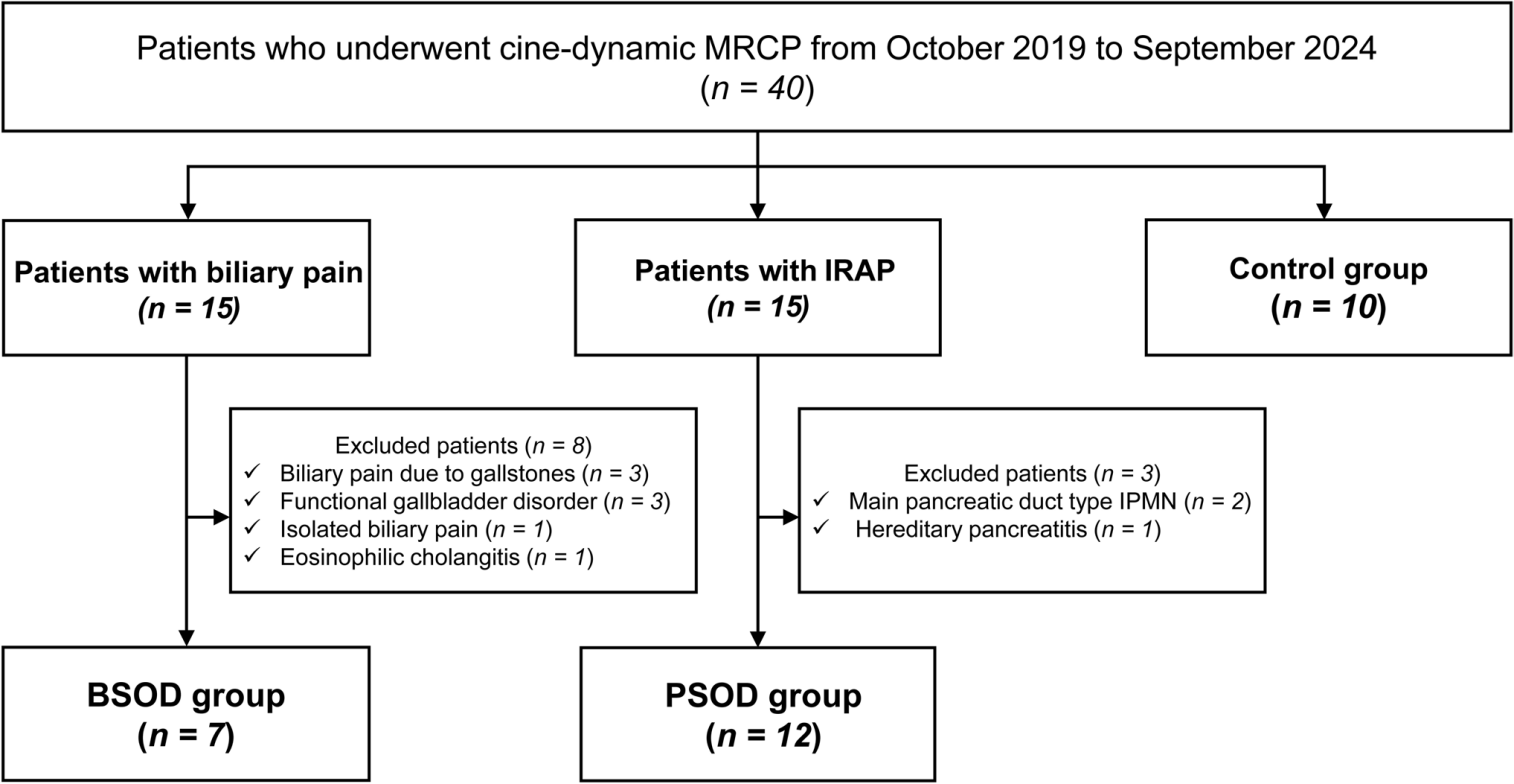
Patient flow diagram. Forty participants were enrolled in the study, including 10 healthy controls. Among patients who underwent cine-dynamic MRCP for biliary pain or suspected IRAP, those ultimately diagnosed with other conditions were excluded. Therefore, the final study cohort comprised 7 patients with suspected BSOD and 12 with suspected PSOD. *MRCP* magnetic resonance cholangiopancreatography, *IRAP* idiopathic recurrent acute pancreatitis, *BSOD* biliary-type sphincter of Oddi dysfunction, *PSOD* pancreatic-type sphincter of Oddi dysfunction

Forty participants were enrolled in the study, including 10 healthy controls. Fifteen patients with biliary pain who underwent cine-dynamic MRCP were initially considered. Among these, three were diagnosed with gallstone-related biliary pain following MRCP, three were found to have functional gallbladder disorder with symptom resolution after cholecystectomy, one experienced biliary pain but did not meet the diagnostic criteria for SOD, and one was diagnosed with eosinophilic cholangitis. These eight patients were excluded, resulting in a final cohort of seven patients with BSOD. Similarly, 15 patients with suspected IRAP underwent cine-dynamic MRCP. Among these, two were later diagnosed with main duct-type intraductal papillary mucinous neoplasm (IPMN) and underwent surgery 1–2 years after MRCP, and one was diagnosed with hereditary pancreatitis based on genetic testing. These three patients were excluded, leaving 12 patients who met the criteria for PSOD (Fig. 1). No patients with chronic pancreatitis were included in the PSOD group.

The baseline characteristics of the control and patient groups are summarized in Table 1. Comparisons were made between the control group and each patient group (BSOD and PSOD). No significant differences were observed in sex distribution, alcohol consumption, or smoking history between groups. However, the patients were significantly older than controls (median age [range]: control, 38 [32–50] years; BSOD, 73 [20–92] years; PSOD, 59 [25–78] years; *p* = 0.003). Although diabetes mellitus and a history of severe acute pancreatitis were observed only in the patient groups, the differences were not statistically significant.

**Table 1:** Patient characteristics

	Total	Control group	BSOD group	PSOD group	*p value*
	*n* = 29	*n* = 10	*n* = 7	*n* = 12	
Median age, years (range)	56 (20–92)	38 (32–50)	73 (20–92)	59 (25–78)	0.003
Sex, male, *n* (%)	11 (37.9)	5 (50)	3 (42.9)	3 (25)	0.50
Alcohol intake (< 50 g/day), present, *n* (%)	8 (37.9)	5 (50)	1 (14.3)	2 (16.7)	0.24
Smoking, present, *n* (%)	12 (41.4)	3 (30)	2 (28.6)	3 (25)	0.62
Diabetes, present, *n* (%)	2 (6.9)	0 (0)	0 (0)	2 (16.7)	0.33
History of severe acute pancreatitis, present, *n* (%)	1 (3.4)	0 (0)	0 (0)	1 (8.3)	0.99
Other medical history, present, *n* (%)	5 (17.2)	0 (0)	2 (28.6)	3 (25)	0.21

*BSOD* biliary-type Sphincter of Oddi dysfunction, *PSOD* pancreatic-type Sphincter of Oddi dysfunction

### Frequency and the secretion grade of bile and pancreatic juice flow assessed using cine-dynamic MRCP

**Fig. 2:**
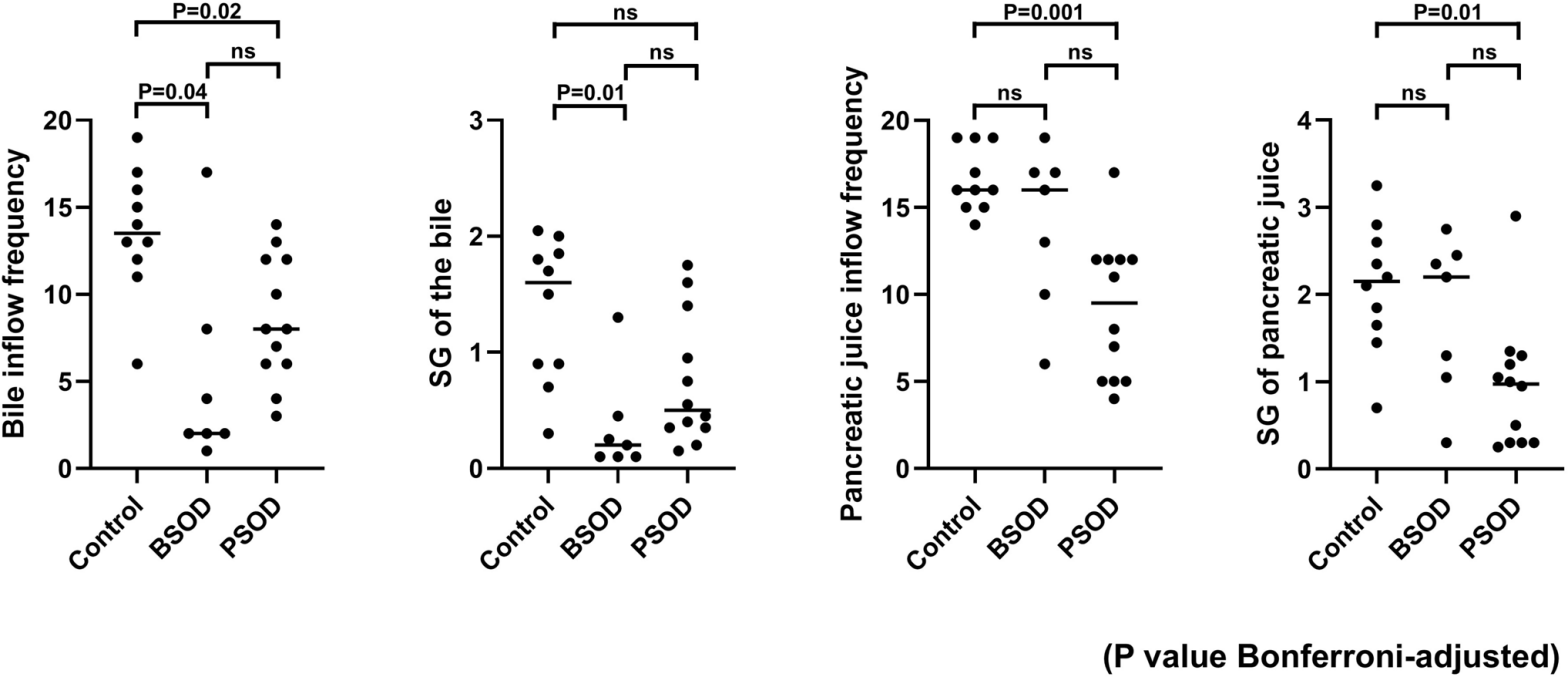
Comparison of the frequency and secretion grade of bile and pancreatic juice flow among the study groups. The frequency and secretion grade of bile and pancreatic juice flow were evaluated and compared across the study groups. Statistical significance was assessed, and *p* values are shown. “ns” indicates non-significant differences

The results for bile and pancreatic juice flow frequency and secretion grade, as measured using cine-dynamic MRCP, are presented in Fig. 2. Inter-observer agreement for bile and pancreatic flow frequency was good (ICC[A,1] = 0.895; 95% CI 0.829–0.936), and agreement for secretion grade was excellent (quadratic weighted *κ* = 0.89). Compared to the control group, both the BSOD and PSOD groups demonstrated significantly lower bile inflow frequency (median, times [range]: control group, 13.5 [6–19] vs. BSOD group, 2.0 [1–17], *p* = 0.04; vs. PSOD group, 8.0 [3–14], *p* = 0.02) and a reduced bile secretion grade in the BSOD group (median [range]: control group, 1.6 [0.3–2.05] vs. BSOD group, 0.2 [0.1–1.3], *p* = 0.01), whereas the difference between the control and PSOD groups was not statistically significant. While no significant difference in the frequency of pancreatic juice inflow was observed between the BSOD and control groups, the PSOD group exhibited a significantly reduced frequency (median, times [range]: control group, 16 [14–19] vs. PSOD group, 9.5 [4–17], *p* = 0.001). Similarly, the secretion grade of pancreatic juice inflow did not differ significantly between the BSOD and control groups, whereas it was significantly lower in the PSOD group (median [range]: control group, 2.15 [0.7–3.25] vs. PSOD group, 0.98 [0.25–2.9], *p* = 0.01). Detailed pairwise comparisons, including exact *p* values, Hodges–Lehmann median differences, and 95% CIs, are provided in Online Resource 9.

### Multivariate linear regression analysis: influence of age on functional imaging outcomes

Multivariate linear regression analysis was conducted to assess the association between the outcome variables (the frequency and secretion grade of bile and pancreatic juice flow) and predictors (age and diagnostic group). The results are presented in Table 2. Across all the models, age was not identified as a significant predictor of any outcome measure. In contrast, the group variable showed a statistically significant association with both the flow frequency and secretion grade. Furthermore, the unstandardized regression coefficients for the group variable closely approximated the differences in median values observed in Fig. 2, thereby supporting the validity of the multivariate models and indicating that group-related differences in flow parameters were not substantially confounded by age. In an age-restricted sensitivity analysis, including only participants aged ≤ 65 years, all cine-dynamic MRCP parameters remained significantly lower in the PSOD group than in control group. Specifically, bile flow frequency (median [IQR]) was 13.5 [12.25–15.75] in control group and 7.5 [6.0–11.5] in PSOD (*p* = 0.006), bile secretion grade was 1.6 [0.9–1.84] vs. 0.5 [0.35–0.90] (*p* = 0.03), pancreatic flow frequency was 16.0 [15.25–18.5] vs. 9.5 [5.5–12.0] (*p* = 0.001), and pancreatic secretion grade was 2.15 [1.70–2.54] vs. 0.98 [0.35–1.28] (*p* = 0.007). Only 2 BSOD patients were aged ≤ 65 years, and therefore, no separate comparison was performed for this subgroup.

**Table 2:** Multivariate linear regression analysis*: influence of age and group on functional imaging outcomes

Predictor	Frequency of bile inflow	SG of bile	Frequency of pancreatic juice inflow	SG of pancreatic juice
Age β, (95% CI), *p* value	− 0.006, (− 0.12–0.11), 0.92	− 0.002, (− 0.017–0.014), 0.82	− 0.03, (− 0.14–0.065), 0.48	− 0.008, (− 0.029–0.014), 0.48
Group (BSOD) β, (95% CI), *p* value	–8.30, (− 13.8 – − 2.86), 0.004	− 0.96, (− 1.68 – − 0.25), 0.01	− 1.62, (− 6.27–3.03), 0.48	− 1.09, (− 1.11–0.89), 0.83
Group (PSOD) β, (95% CI), *p* value	− 4.91, (− 9.24 – − 0.59), 0.03	− 0.59, (− 1.17 – − 0.03), 0.04	− 6.80, (− 10.5 – − 3.10), < 0.001	− 1.01, (− 1.80 – − 0.21), 0.02

*Β* unstandardized regression coefficients, *BSOD* biliary-type Sphincter of Oddi dysfunction, *PSOD* pancreatic-type Sphincter of Oddi dysfunction, *SG* secretion grade

^*^Multiple linear regression analysis was performed to assess whether cine-dynamic MRCP outcomes were influenced by age and group differences

The relationship between each parameter and age is shown in Online Resource 10.

### Therapeutic effect of EST assessed using cine-dynamic MRCP

**Fig. 3:**
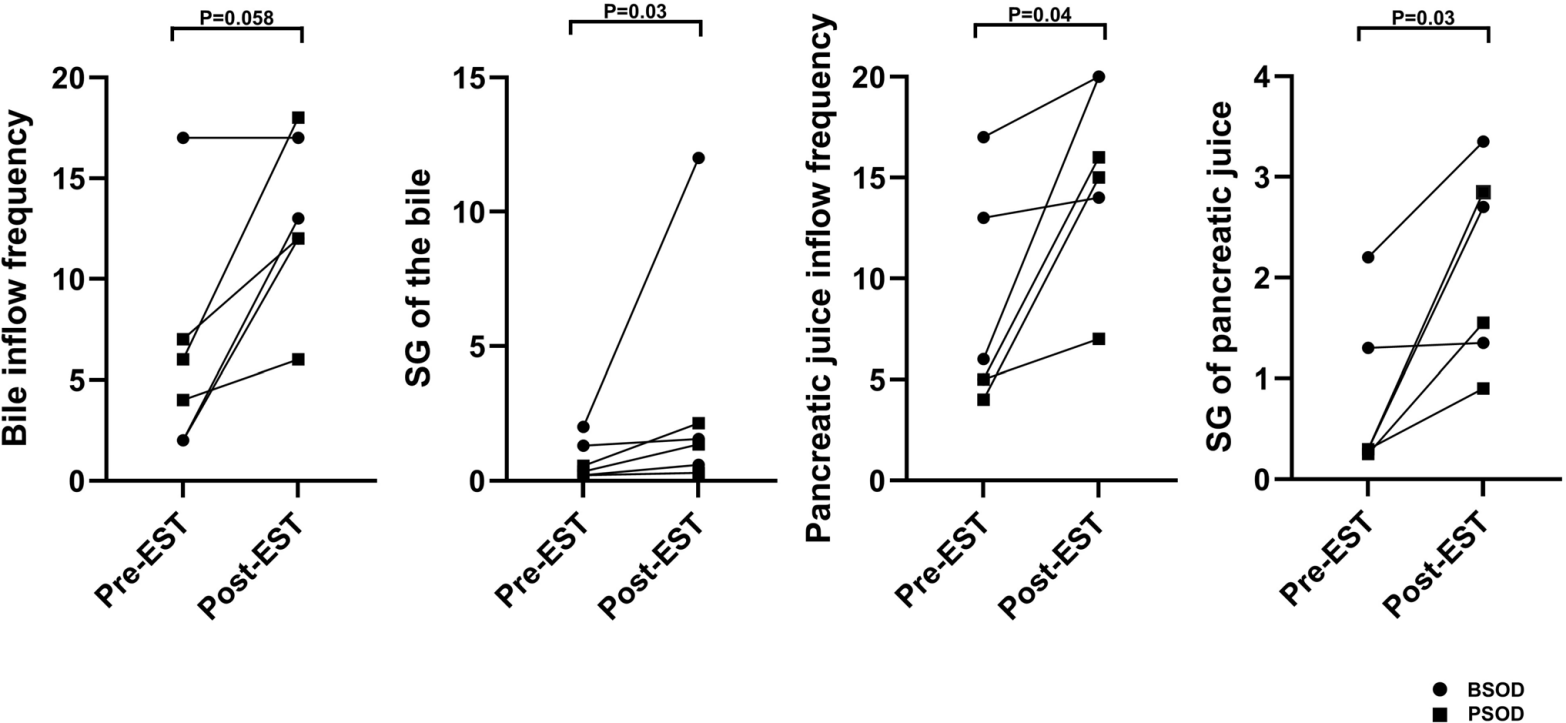
Therapeutic effect of EST assessed using cine-dynamic MRCP. Changes in bile and pancreatic juice flow parameters before and after EST as assessed using cine-dynamic MRCP are shown. Flow frequency and secretion grade were evaluated in 7 patients with suspected sphincter of Oddi dysfunction (3 with biliary-type SOD [BSOD] and 4 with pancreatic-type SOD [PSOD]); among them, 6 patients underwent cine-dynamic MRCP both before and after EST and were included in this analysis. Paired dot plots display pre and post-EST values for bile and pancreatic juice flow frequency and secretion grade, with each line representing an individual patient. Closed circles indicate patients with BSOD, and closed squares indicate those with PSOD. *SOD* sphincter of Oddi dysfunction, *BSOD* biliary-type SOD, *PSOD* pancreatic-type SOD, *EST* endoscopic sphincterotomy, *MRCP* magnetic resonance cholangiopancreatography, *SG* secretion grade

Seven patients who underwent cine-dynamic MRCP subsequently underwent EST to prevent the recurrence of biliary pain or pancreatitis. Among them, 3 patients were classified into the BSOD group and 4 into the PSOD group. Among these 7 patients, 6 underwent follow-up cine-dynamic MRCP. The median interval between EST and follow-up cine-dynamic MRCP was 26 days (range, 11–54 days). While a few patients showed minimal changes in the frequency of bile or pancreatic juice flow before and after EST, the majority demonstrated notable increases in both flow frequency and secretion grade following the procedure. To quantitatively assess these changes, the Wilcoxon signed-rank test was performed for each parameter—bile inflow frequency, secretion grade of bile, pancreatic juice inflow frequency, and secretion grade of pancreatic juice—in patients who underwent EST. The resulting *p* values were 0.058, 0.03, 0.04, and 0.03, respectively. A paired dot (spaghetti) plot illustrating individual pre and post-EST changes has been added in Fig. 3. A representative case of post-EST improvement is presented in Online Resource 11 and 12. No procedure-related adverse events such as PEP or bleeding were observed. The median follow-up duration was 18 months (range, 13–20 months), and no patient was lost to follow-up. Importantly, none of the patients experienced a recurrence of biliary pain or pancreatitis during the follow-up period, which exceeded 1 year.

## Discussion

To the best of our knowledge, this is the first study to evaluate SO function using cine-dynamic MRCP. Compared with healthy controls, patients with suspected BSOD and PSOD demonstrated significantly reduced bile and pancreatic juice flow frequencies and lower secretion grades, respectively. These findings suggest that impaired bile and pancreatic juice dynamics reflect underlying SOD. Furthermore, in patients who underwent EST, both bile and pancreatic juice flow parameters improved after the procedure, supporting the utility of cine-dynamic MRCP as a functional imaging tool for SO assessment.

In the PSOD group, both the frequency and secretion grade of bile and pancreatic juice flow were significantly lower than those in healthy controls. This dual impairment is consistent with the findings of previous SOM-based studies reporting that approximately 30–40% of patients exhibit elevated basal pressure in both the biliary and pancreatic sphincters [10, 11]. These findings further support the potential of cine-dynamic MRCP as a non-invasive surrogate for SOM in the comprehensive assessment of SOD.

Cine-dynamic MRCP is a non-invasive and straightforward technique that requires only a few breath-hold acquisitions in addition to conventional MRCP. Its minimal operator dependence and broad feasibility in standard clinical MRI settings make it highly practical. In addition, the ability to visually capture and quantitatively compare dynamic changes before and after treatment yields intuitive results that can enhance both clinical assessment and patient understanding. Collectively, these features underscore the practicality and accessibility of cine-dynamic MRCP as a valuable tool for evaluating SO function and supporting patient education about SOD.

Diagnosing both PSOD and BSOD requires the exclusion of structural diseases, necessitating conventional MRCP. By incorporating cine-dynamic sequences into routine MRCP protocols, sphincter function can be evaluated non-invasively, eliminating the need for additional tests, such as scintigraphy, which involves radiation exposure, prolonged examination time, and costly reagents. Importantly, cine-dynamic MRCP does not carry the risk of PEP associated with endoscopic investigations, including SOM. Cine-dynamic MRCP offers an efficient, radiation-free, and widely implementable alternative for functional assessment at MRI-equipped facilities.

This study had some limitations. First, this was a single-center study with a relatively small sample size. However, considering the rarity of SOD in Japan, evaluating a large cohort remains inherently difficult. Second, none of the patients underwent concurrent SOM. Given the invasive nature of SOM, its association with PEP, and the challenges of obtaining devices for sphincter pressure measurement, performing SOM remains difficult in Japan. We did not compare cine-dynamic MRCP with SOM or hepatobiliary scintigraphy in this pilot study; therefore, criterion validity cannot yet be inferred. Third, the possibility of undetected neoplastic disease cannot be ruled out. For example, 2 cases excluded from the IRAP group were later diagnosed with main-duct IPMN following cine-dynamic MRCP, EST, and subsequent follow-up. Both patients experienced recurrent pancreatitis and significant main pancreatic duct dilation within 1 year. These findings underscore the importance of being vigilant for neoplastic lesions in similar cases. Fourth, the mean age of patients was higher than that of controls. Although age was not a significant predictor in the multivariate linear regression analysis, all volunteers were aged 50 years or younger. As the pancreatic juice flow frequency and secretion grade decline with age [15], the potential for age-related confounding effects remains, particularly in cases of PSOD. To further minimize age-related confounding, a sensitivity analysis limited to participants aged ≤ 65 years was conducted, which confirmed that cine-dynamic MRCP parameters remained significantly reduced in PSOD patients compared with controls. Despite these limitations, this study demonstrated that cine-dynamic MRCP has the potential to serve as an innovative, objective, and minimally invasive diagnostic tool for SOD. Future large-scale studies are warranted to validate its clinical utility and to establish standardized diagnostic criteria.

In conclusion, cine-dynamic MRCP shows promise as a noninvasive alternative to SOM for evaluating sphincter function and warrants validation in larger, multicenter cohorts.

## Supplementary Information

Below is the link to the electronic supplementary material.

Supplementary file1 (TIF 735 KB)

Supplementary file2 (TIF 606 KB)

Supplementary file3 (TIF 595 KB)

Supplementary file4 (MP4 623 KB)

Supplementary file5 (DOCX 18 KB)

Supplementary file6 (DOCX 2582 KB)

Supplementary file7 (TIF 827 KB)

Supplementary file8 (TIF 4513 KB)

Supplementary file9 (DOCX 19 KB)

Supplementary file10 (TIF 594 KB)

Supplementary file11 (MP4 692 KB)

Supplementary file12 (MP4 653 KB)
