# Supplementary figures and images for: Prospective pilot study of functional assessment of the Sphincter of Oddi via cine-dynamic MRCP with selective inversion recovery pulse
Source: J Gastroenterol. 2026 Jan 22;61(4):487–95. doi: 10.1007/s00535-026-02344-1 (PMC13048933; doi:10.1007/s00535-026-02344-1)

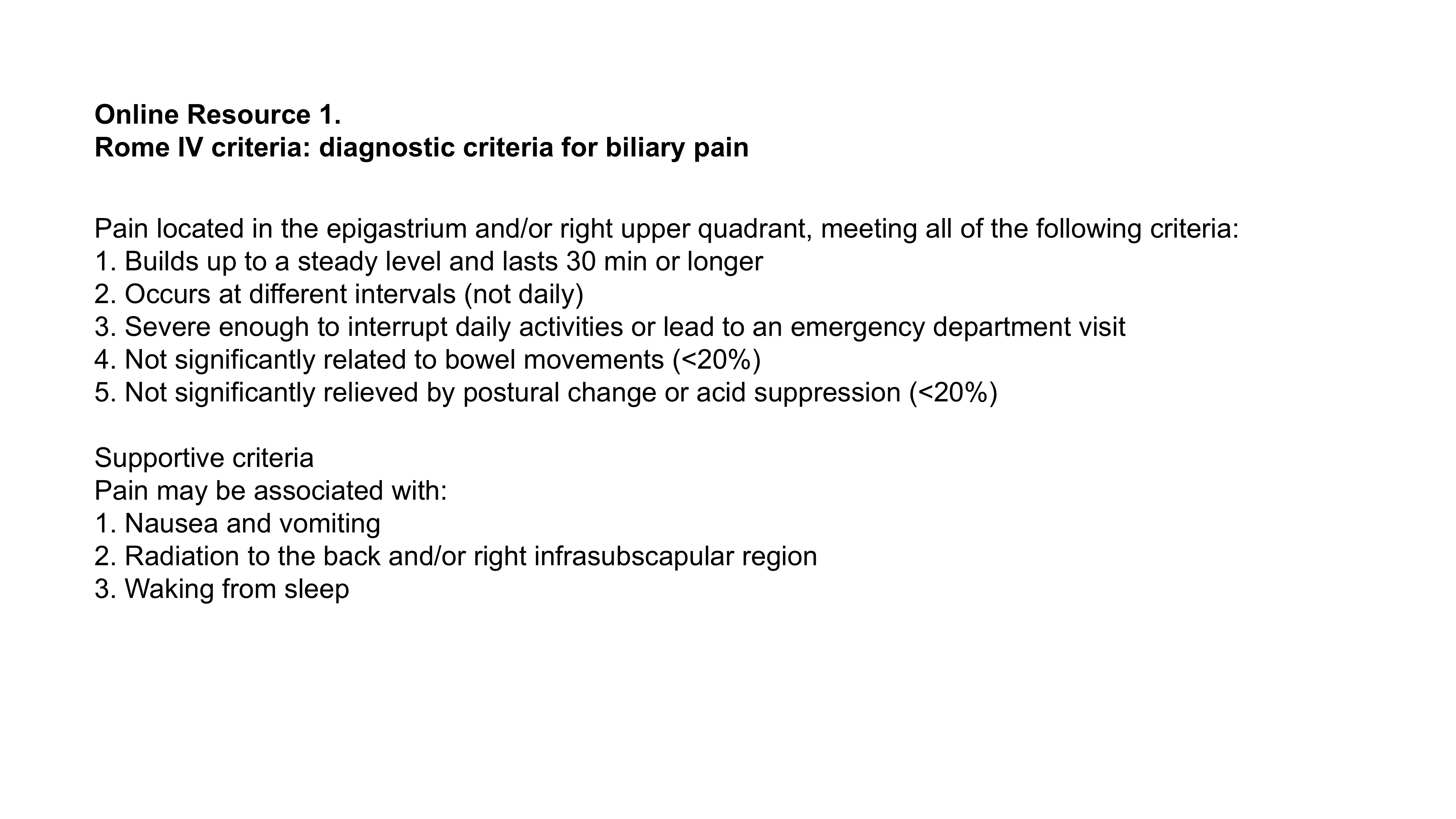

Supplement: Supplementary file 1 — Supplementary file1 (TIF 735 KB) [file 535_2026_2344_MOESM1_ESM.tif]

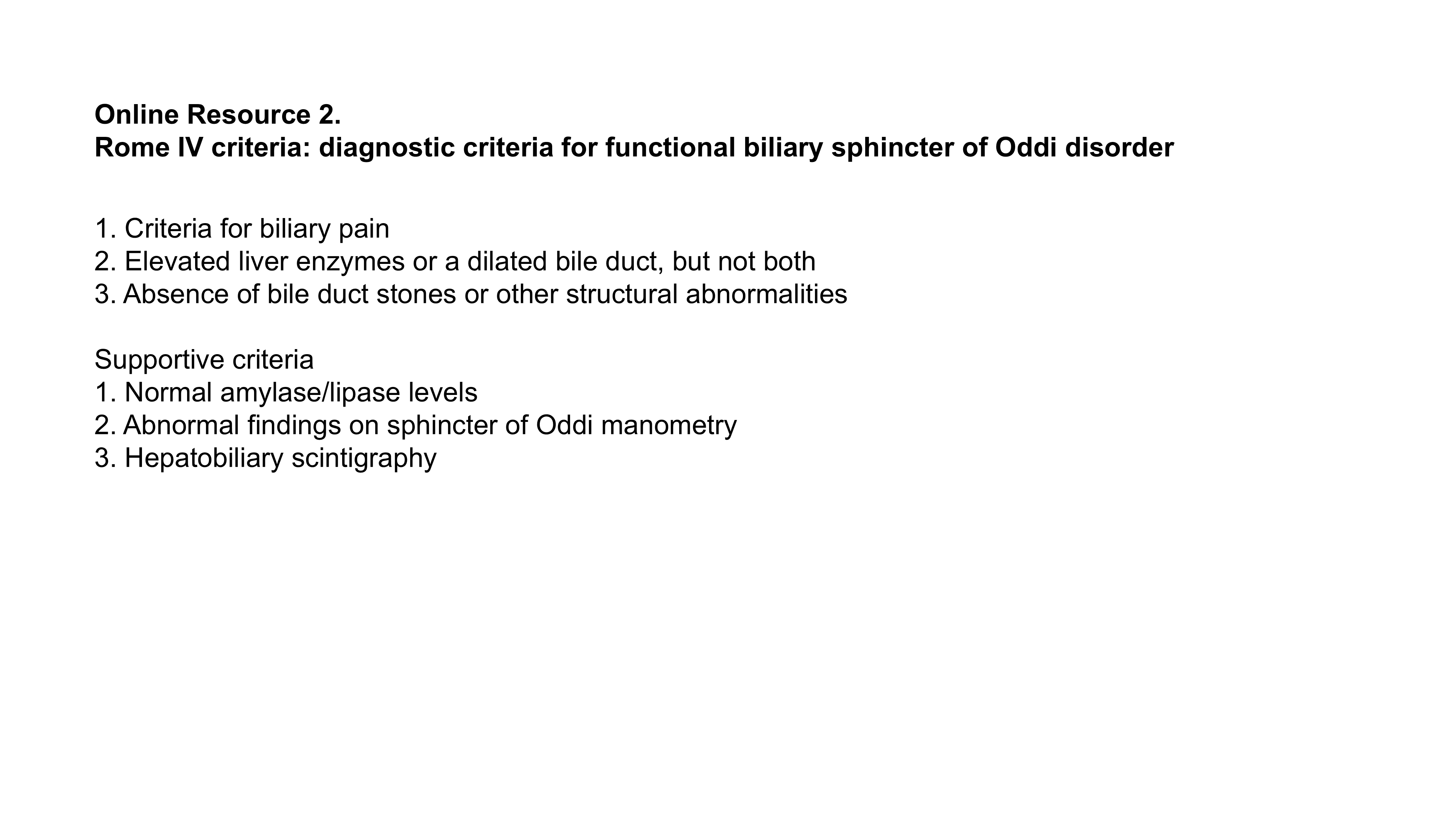

Supplement: Supplementary file 2 — Supplementary file2 (TIF 606 KB) [file 535_2026_2344_MOESM2_ESM.tif]

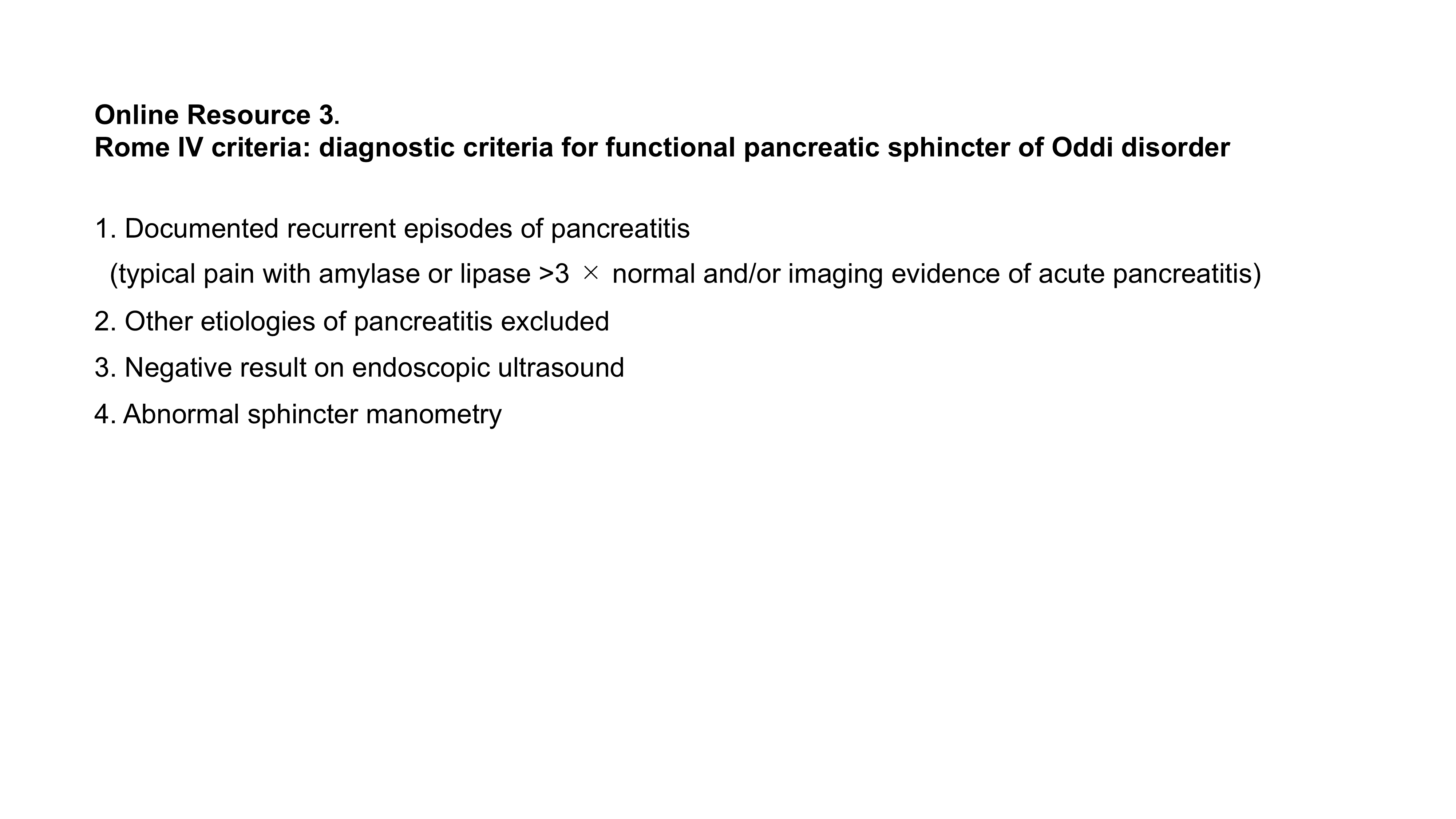

Supplement: Supplementary file 3 — Supplementary file3 (TIF 595 KB) [file 535_2026_2344_MOESM3_ESM.tif]

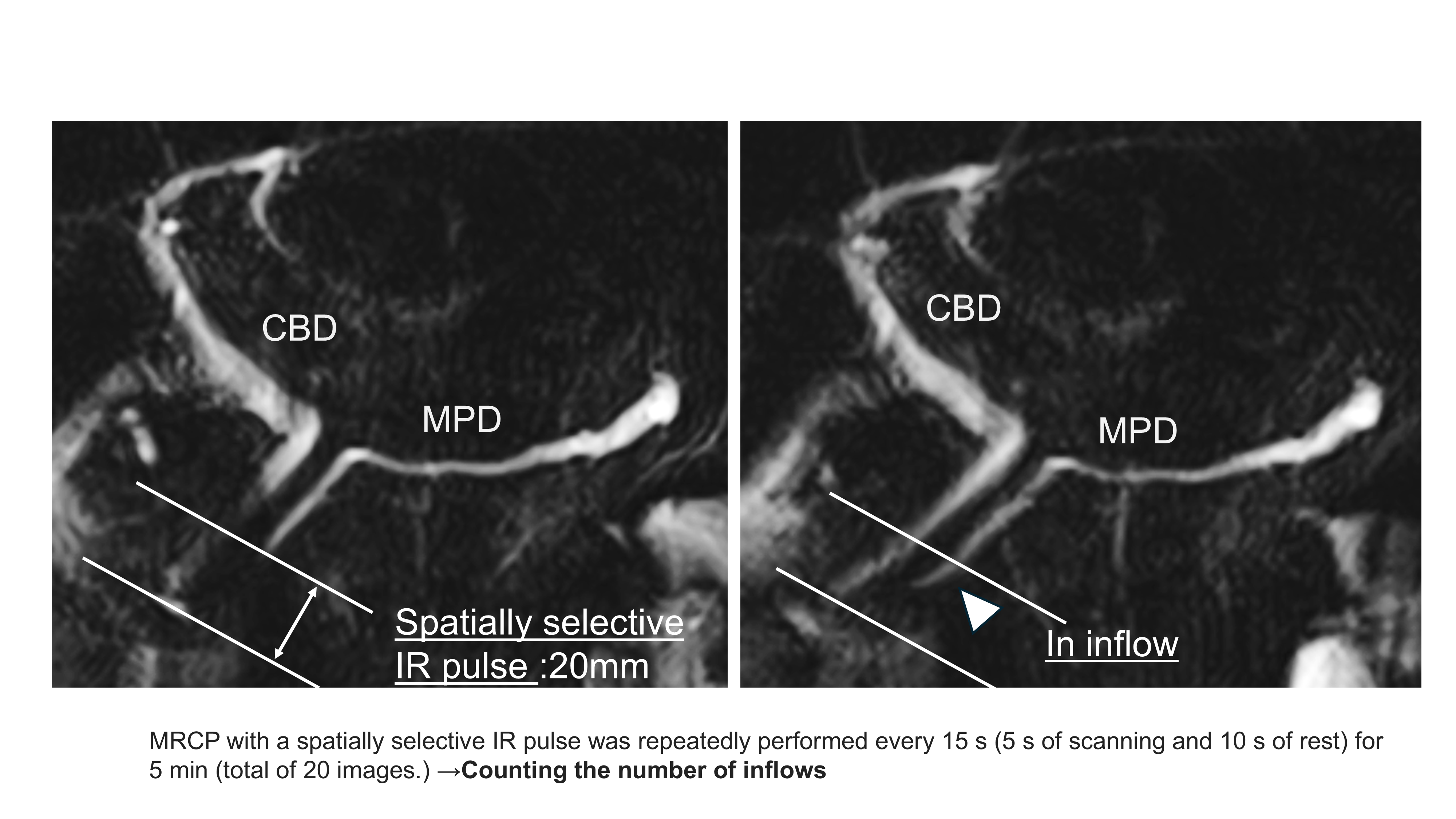

Supplement: Supplementary file 6 — Supplementary file6 (DOCX 2582 KB) [file 535_2026_2344_MOESM6_ESM.tif]

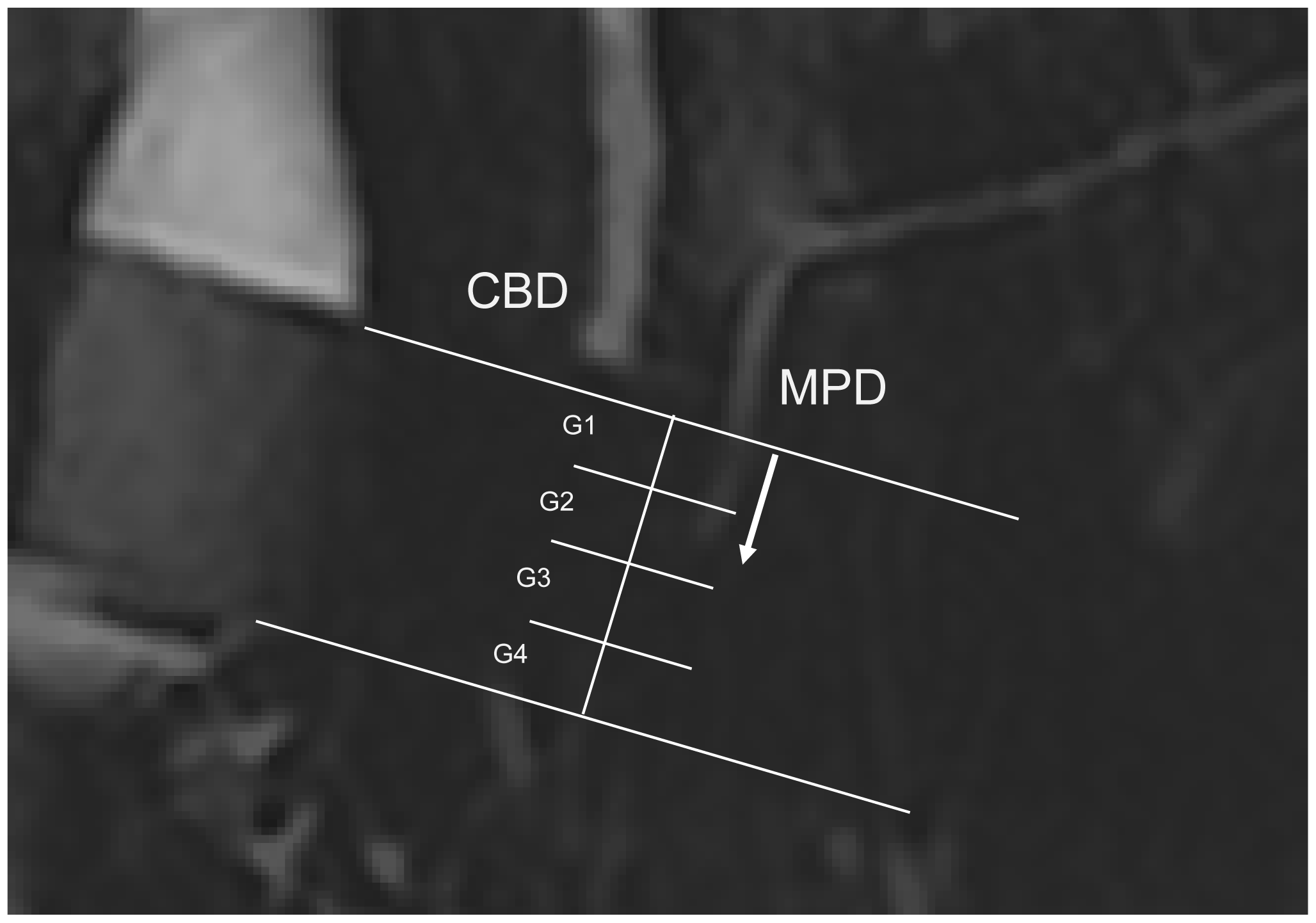

Supplement: Supplementary file 7 — Supplementary file7 (TIF 827 KB) [file 535_2026_2344_MOESM7_ESM.tif]

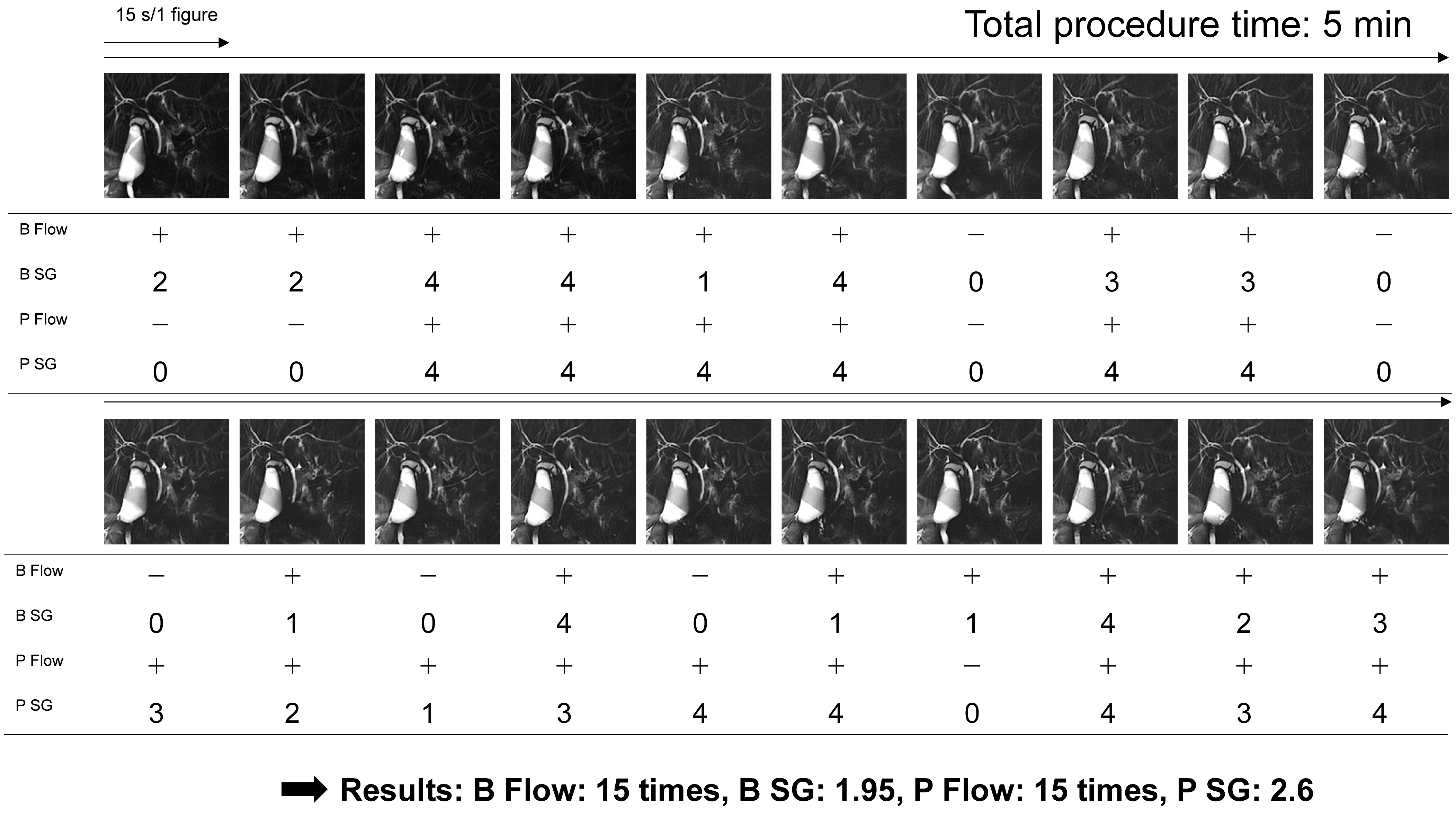

Supplement: Supplementary file 8 — Supplementary file8 (TIF 4513 KB) [file 535_2026_2344_MOESM8_ESM.tif]

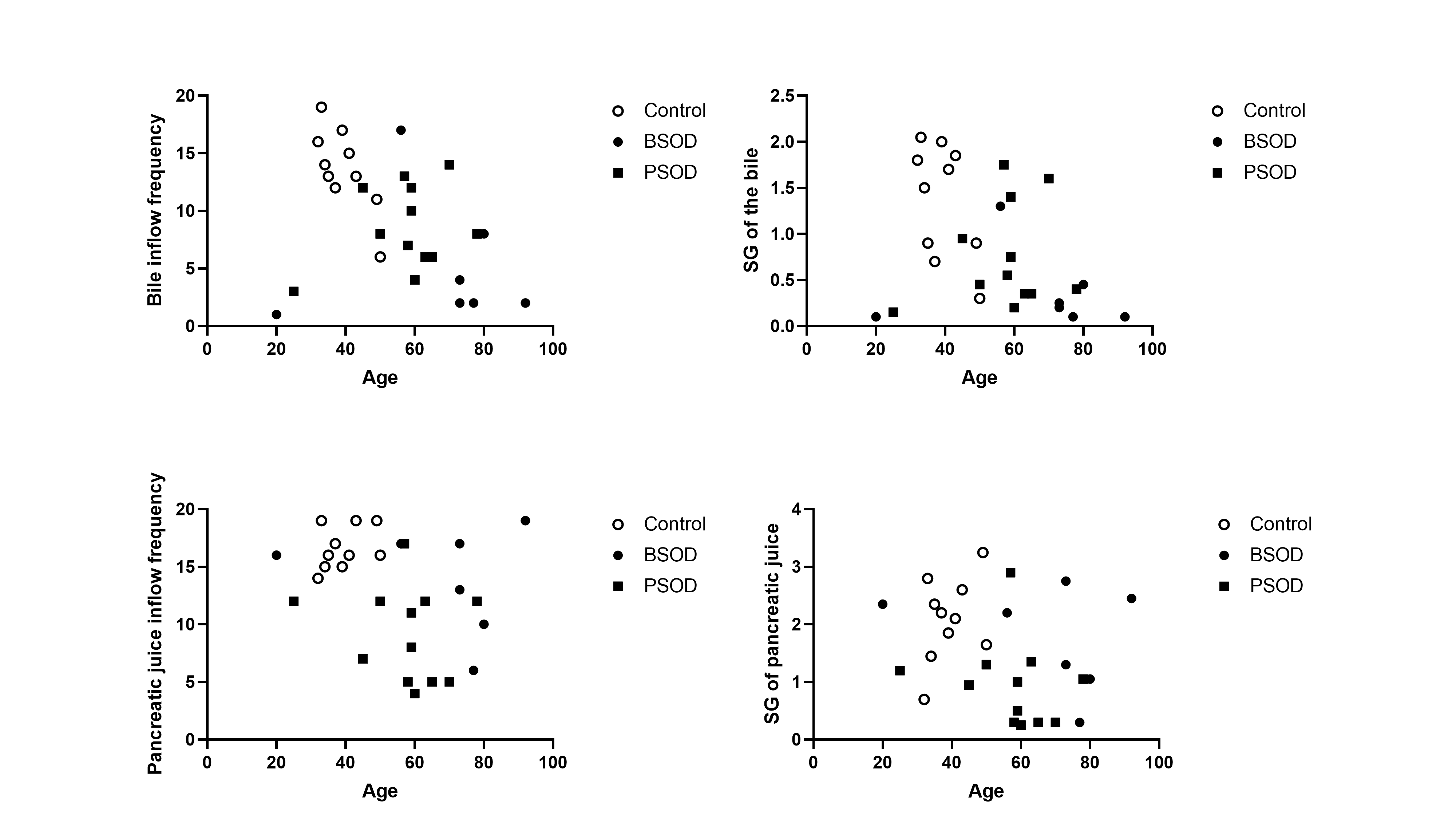

Supplement: Supplementary file 10 — Supplementary file10 (TIF 594 KB) [file 535_2026_2344_MOESM10_ESM.tif]
