# Supplementary material for: Prospective pilot study of functional assessment of the Sphincter of Oddi via cine-dynamic MRCP with selective inversion recovery pulse
Source: J Gastroenterol. 2026 Jan 22;61(4):487–95. doi: 10.1007/s00535-026-02344-1 (PMC13048933; doi:10.1007/s00535-026-02344-1)
Supplement: Supplementary file 5 — Supplementary file5 (DOCX 18 KB) [file 535_2026_2344_MOESM5_ESM.docx]

**Online Resource 5. Summarizes the cine-dynamic MRCP acquisition parameters and the minimum technical requirements for reproducibility across MRI systems.**

| **Parameter** | **Setting / Requirement** | **Notes** |
| --- | --- | --- |
| **MRI system** | 3.0 T (Vantage Centurian; Canon Medical Systems, Tochigi, Japan) | Equivalent performance expected with other 3.0 T platforms |
| **Coils** | 16-channel anterior phased-array coil and 32-channel bed-embedded spine coil | Standard multi-channel setup sufficient |
| **Breath-hold duration per acquisition** | 5 seconds | Performed at end-expiration |
| **Rest interval between scans** | 10 seconds | Passive breathing |
| **Total dynamic acquisitions** | 20 frames (5 minutes in total) | Repeated every 15 seconds |
| **Trigger mode** | Pulse-wave synchronization (used only for selective IR pulse timing) | ECG or peripheral gating not required |
| **Spatially selective IR pulse** | Width 20 mm; inversion time 2200 ms; flip angle 240° | Positioned just above the papilla, covering the distal common bile duct and main pancreatic duct |
| **Acquisition time per IR pulse** | 5 seconds | Single breath-hold acquisition |
| **Repetition / echo time (TR/TE)** | 5000/500 ms | Comparable timing feasible on most 3.0 T scanners |
| **Field of view / Matrix** | 32 × 32 cm / 320 × 320 | In-plane resolution approximately 1 mm |
| **Slice thickness** | 40 mm (thick-slab) | Oblique coronal orientation |
| **Fat suppression** | SPAIR (spectral attenuated inversion recovery) | Any comparable fat suppression method acceptable |
| **Parallel imaging factor** | 2 | Optional |
| **Reconstruction method** | Deep-learning reconstruction (AiCE) | Optional; standard reconstruction acceptable |
| **Minimum technical requirements** | 3.0 T field strength, selective IR capability, thick-slab 2D MRCP sequence, and ≤5-second breath-hold | Required for reproducibility across vendors |
| **Scan time summary** | 20 acquisitions (5 seconds each) with 10-second rest intervals, totaling approximately 5 minutes of dynamic scanning |  |
