# Supplementary material for: Prospective pilot study of functional assessment of the Sphincter of Oddi via cine-dynamic MRCP with selective inversion recovery pulse
Source: J Gastroenterol. 2026 Jan 22;61(4):487–95. doi: 10.1007/s00535-026-02344-1 (PMC13048933; doi:10.1007/s00535-026-02344-1)
Supplement: Supplementary file 9 — Supplementary file9 (DOCX 19 KB) [file 535_2026_2344_MOESM9_ESM.docx]

**Online Resource 9. Pairwise effect sizes for cine-dynamic MRCP indices.**

| **Outcome** | **Pairwise comparison** | **P Value** | **HL, median difference (95% CI)** |
| --- | --- | --- | --- |
| Bile inflow frequency | Control vs BSOD | 0.04 | +10.0 (+2.0 – +14.0) |
|  | Control vs PSOD | 0.02 | +5.0 (+1.0 – +9.0) |
|  | BSOD vs PSOD | 0.19 | -4.0 (-10.0 – +3.0) |
| Secretion grade of the bile | Control vs BSOD | 0.01 | +1.05 (+0.20 – +1.75) |
|  | Control vs PSOD | 0.08 | +0.55 (-0.05 – +1.45) |
|  | BSOD vs PSOD | 0.14 | -0.30 (-1.30 – +0.10) |
| Pancreatic juice inflow frequency | Control vs BSOD | 1.00 | +2.0 (-2.0 – +9.0) |
|  | Control vs PSOD | 0.001 | +7.0 (+4.0 – +11.0) |
|  | BSOD vs PSOD | 0.11 | +5.0 (-1.0 – +12.0) |
| Secretion grade of pancreatic juice | Control vs BSOD | 1.00 | +0.26 (-0.80 – +1.55) |
|  | Control vs PSOD | 0.01 | +1.25 (+0.40 – +1.95) |
|  | BSOD vs PSOD | 0.22 | +1.0 (-0.25 – +1.95) |

Abbreviations: HL, Hodges–Lehmann; CI, confidence interval; BSOD, biliary-type sphincter of Oddi dysfunction; PSOD, pancreatic-type sphincter of Oddi dysfunction.
